# Epidemiology of Early-Onset Colorectal Cancer: A Systematic Review and Meta-Analysis of Incidence, Temporal Trends, and Associated Factors

**DOI:** 10.7759/cureus.110916

**Published:** 2026-06-15

**Authors:** Minhaz Ahmad, Abdul Subhan Talpur, Zeeshan Solangi, Hammad Qadri, Fawad Talat, Nadeem Chauhan, Fatima Becirovic, Audery Roosevelt, Mahnoor Azeem, Adila Tabassum, Muhammad Junaid Mahboob, Hamza Usman, Sardar Muhammad Naseer-Ud-Din, Aisha Siddiqui, Lal Muhammad, Abdelhamid Ben Selma, Toseef Javaid

**Affiliations:** 1 Internal Medicine, United Health Services (UHS) Wilson Medical Center, Johnson City, USA; 2 Internal Medicine, Yale School of Medicine, New Haven, USA; 3 Gastroenterology and Hepatology, United Health Services (UHS) Wilson Medical Center, Johnson City, USA; 4 Medicine and Surgery, Lala Lajpat Rai Memorial Medical College, Meerut, IND; 5 Health Sciences, Oklahoma State University, Tulsa, USA; 6 Medicine, State University of New York (SUNY) Broome Community College, Binghamton, USA; 7 Medicine, State University of New York (SUNY) Upstate Medical University, Syracuse, USA; 8 Medicine and Surgery, Ziauddin University, Karachi, PAK; 9 Internal Medicine, Fatima Jinnah Medical University, Lahore, PAK; 10 Internal Medicine, State University of New York (SUNY) Upstate Medical University, Syracuse, USA; 11 Medicine, Wayne State University School of Medicine, Detroit, USA; 12 Gastroenterology and Hepatology, Arnot Ogden Medical Center, Elmira, USA

**Keywords:** early-onset colorectal cancer, incidence, risk factors, temporal trends, young onset

## Abstract

Early-onset colorectal cancer (EOCRC) refers to colorectal cancer (CRC) diagnosed in adults younger than 50 years. We conducted this systematic review and meta-analysis to estimate EOCRC incidence, evaluate temporal trends from 1990 to 2021, identify factors associated with increased EOCRC risk, and project the anticipated burden through 2030. PubMed/MEDLINE, Embase, and the Cochrane Central Register of Controlled Trials (CENTRAL) were searched from inception through December 31, 2025. Population-based registry studies, cohort studies, and case-control studies reporting EOCRC incidence, temporal trends, or associated factors were included. The protocol was prospectively registered in the International Prospective Register of Systematic Reviews (PROSPERO) (CRD420261345410). Two reviewers independently screened records, extracted data, and assessed study quality using the Newcastle-Ottawa Scale. Certainty of evidence was assessed using the Grading of Recommendations Assessment, Development, and Evaluation (GRADE) framework for the primary incidence estimate and the leading modifiable associated factors. Incidence rates and adjusted association estimates were pooled using random-effects meta-analysis, with analyses stratified by study design where appropriate. Prespecified subgroups included geographic region, sex, tumor subsite, Human Development Index stratum, calendar period, and age group; temporal trends were examined using meta-regression. Fifty-two studies from 52 countries were included. The pooled incidence showed substantial geographic variation, with the highest rates in Australasia and North America and the lowest rates in sub-Saharan Africa. Incidence increased significantly over time, with the Western Pacific showing the fastest rise. Rectal cancer increased more steeply than colon cancer. Family history of CRC and inflammatory bowel disease showed the strongest non-modifiable associations. Among modifiable exposures, sugar-sweetened beverages, processed meat consumption, obesity, Western dietary pattern, smoking, and alcohol use were associated with higher EOCRC odds. On current trajectories, EOCRC is projected to represent a growing share of CRC diagnoses by 2030. EOCRC is increasing across diverse geographic settings. These findings support clinical vigilance for alarm symptoms in younger adults, risk-stratified prevention, and public health strategies targeting modifiable lifestyle exposures.

## Introduction and background

Colorectal cancer (CRC) remains the third most commonly diagnosed malignancy worldwide and the second most common cause of cancer-related mortality, with approximately 1.93 million new cases and 940,000 deaths recorded in 2022 alone [1-3]. For most of the 20th century, CRC was considered a disease of older adulthood, and screening programs were built around that premise. That picture has changed substantially. In many high-income countries, colonoscopy-based screening has successfully reduced incidence and mortality among people over 50, yet rates in younger adults have moved in precisely the opposite direction. This rise in CRC diagnosed before the age of 50, now widely called early-onset colorectal cancer (EOCRC), has become one of the most discussed and least fully explained trends in gastrointestinal oncology [4-6].

The pace of change is striking. In the United States, incidence among adults aged 20-34 years has climbed by roughly 2.4% per year since 2010, while the 35-49-year age group has followed at about 1.7% annually [7,8]. Similar patterns have now been reported across Europe and the Asia-Pacific region and, more recently, in countries once considered low incidence, including parts of Eastern Europe, Latin America, and Asia [5,9]. A 2024 American Cancer Society analysis drawing on data from 50 countries found rising EOCRC rates in 27 of them with the increase either confined to younger adults or outpacing the trend in older cohorts in 20 of those countries [10].

Despite the growing concern, EOCRC has not yet been characterized comprehensively across diverse geographic settings using a framework that separates incidence, temporal trends, exposure associations, and burden projections. Prior reviews have often been limited by geographic scope, restricted calendar periods, or isolated assessment of incidence without the integrated evaluation of temporal trends and associated exposures [5,6,10,11]. EOCRC also differs clinically and biologically from late-onset CRC: younger patients more commonly present with rectal tumors, are often diagnosed at an advanced stage, and may show distinct molecular and microbiome-related features that could influence tumor behavior and treatment response [11,12]. However, reported increases in EOCRC incidence must be interpreted cautiously. Observed trends may reflect a combination of true disease burden, birth-cohort effects, changes in early-life exposures, increasing obesity and metabolic risk, diet and microbiome shifts, greater diagnostic intensity, colonoscopy access, and improved cancer registry capture [11-15]. Therefore, distinguishing biological increase from detection-related effects remains a central methodological challenge in EOCRC epidemiology.

Understanding what is driving EOCRC matters for several practical reasons. First, most national guidelines still recommend routine screening from ages 45 to 50; the United States lowered its threshold from 50 to 45 in 2018, but most EOCRC cases still fall outside any organized screening program. As a result, most patients are diagnosed only after developing symptoms, by which point the disease is frequently at an advanced stage [13-17]. Second, many of the exposures driving EOCRC, i.e., diet, physical activity, body weight, and alcohol use, are modifiable, meaning that primary prevention is feasible if the evidence is clear enough to act on. Third, modelling suggests that without intervention, EOCRC will represent 11% of colon cancers and 23% of rectal cancers globally by 2030 [17-23], placing a substantial and growing strain on health systems worldwide, particularly given that EOCRC strikes during the most economically and socially productive decades of life [19,24].

Against this backdrop, we conducted an updated systematic review and meta-analysis with four objectives: to estimate pooled regional EOCRC incidence; to characterize temporal trends using average annual percent change (AAPC), defined as the average yearly percentage change in incidence over a defined time interval; to quantify factors associated with increased EOCRC risk; and to project the anticipated burden through 2030. Age-standardized incidence refers to incidence adjusted to a standard age distribution, allowing more comparable estimates across populations with different age structures.

## Review

Methods

Study Registration and Reporting

This review was planned, conducted, and reported in accordance with the Preferred Reporting Items for Systematic Reviews and Meta-Analyses (PRISMA) 2020 statement for systematic reviews and meta-analyses [25]. The protocol was prospectively registered in the International Prospective Register of Systematic Reviews (PROSPERO) (CRD420261345410) before record screening. Any deviations from the registered protocol were reviewed and documented before final synthesis.

Search Strategy

Working with a medical librarian, we searched PubMed/MEDLINE, Embase, and the Cochrane Central Register of Controlled Trials (CENTRAL) from inception through December 31, 2025, combining Medical Subject Headings (MeSH) with free-text keywords. The core search terms included the following: "colorectal cancer", "colon cancer", "rectal cancer", "colorectal neoplasm", "early-onset", "young-onset", "young adults", "age of onset", "incidence", "prevalence", "epidemiology", "temporal trends", "associated factors", "global burden", and related variants (Appendix A). We also hand-searched reference lists of all included papers and performed forward citation tracking on high-yield articles to avoid missing relevant work not captured electronically. Grey literature and population-level data sources, including the Global Cancer Observatory (GLOBOCAN), Global Burden of Disease (GBD), and Cancer Incidence in Five Continents, were consulted for contextual epidemiologic and burden estimates but were not pooled directly with primary observational registry estimates in the main incidence meta-analysis. Preprint servers, including medRxiv, were not searched because the review was restricted to peer-reviewed publications and established epidemiologic data sources. Conference abstracts were excluded unless sufficient full-text peer-reviewed data were available. Studies published in any language were eligible, provided reliable EOCRC-specific data extraction was possible.

Eligibility Criteria

Inclusion criteria: We included the following: (1) population-based studies, national or regional cancer registry analyses, prospective or retrospective cohort studies, and case-control studies reporting the incidence, prevalence, or factors associated with CRC risk in individuals aged <50 years; (2) studies providing data from any geographic region; (3) studies published in any language; and (4) studies published between January 1, 1990, and December 31, 2025.

Exclusion criteria: We excluded the following: (1) case reports, editorials, letters, conference abstracts, and expert opinion pieces; (2) studies focused exclusively on hereditary CRC syndromes such as familial adenomatous polyposis or Lynch syndrome without separately reporting sporadic EOCRC; (3) studies with sample sizes fewer than 100 EOCRC cases; (4) studies where age groups could not be separated to isolate the <50 cohort; and (5) duplicate publications reporting the same cohort without adding new data. The threshold of at least 100 EOCRC cases was prespecified to reduce statistical instability from sparse incidence estimates, minimize undue influence of highly imprecise small-study estimates on pooled results, and improve comparability across heterogeneous population settings [26]. We acknowledge that this criterion may have excluded informative studies from underrepresented or low-resource regions; therefore, the potential impact of excluding small studies was considered in sensitivity and narrative analyses.

Included studies were classified a priori according to their primary analytic contribution: incidence estimation, temporal trend analysis, exposure-association analysis, or burden projection. Population-based registry studies were used primarily for incidence and temporal trend synthesis. Cohort and case-control studies were used primarily for exposure-association analyses. GBD and other modelled burden datasets were treated as secondary modelled estimates and were used for contextual trend interpretation and projection modelling rather than being pooled directly with primary observational registry estimates. This approach was used because modelled health estimates synthesize multiple data sources and assumptions and require transparent reporting distinct from primary observational studies [27].

Study Selection

Two reviewers worked independently and in parallel throughout, using Covidence (Veritas Health Innovation, Melbourne, Australia) to manage the screening process. Titles and abstracts were assessed first, and records meeting eligibility criteria were advanced to full-text review by the same reviewer pair. When multiple reports used overlapping registry populations, identical national datasets, or partially overlapping calendar periods, we retained the report with the most complete EOCRC-specific dataset, longest study period, largest analyzable sample, most detailed subgroup reporting, and clearest incidence or exposure-association estimates. Duplicate reports were excluded unless they contributed unique non-overlapping subgroup, temporal, geographic, or exposure-association data. To avoid double-counting, GBD and other modelled burden estimates were not treated as independent primary registry datasets when overlapping registry-derived incidence data were already included; instead, they were used for contextual trend interpretation and projection modelling. Although studies in any language were eligible, reliable EOCRC-specific data extraction was required. Disagreements at either screening stage were resolved by discussion, and unresolved conflicts were adjudicated by a third senior reviewer. Agreement at the full-text stage was substantial (Cohen's κ = 0.82). A formal title/abstract κ statistic was not retained by the screening platform; however, disagreements at the title/abstract stage were resolved by consensus, with unresolved conflicts adjudicated by a third senior reviewer.

Data Extraction

Data were extracted onto standardized forms that had been piloted and refined before use (Appendix B). One reviewer completed the initial extraction, and a second reviewer independently verified every extracted item against the source article. Discrepancies were resolved through discussion, and unresolved disagreements were adjudicated by a third senior reviewer. Extracted variables included author, year, country, study design, data source, age cut-off, study period, sample size, EOCRC case numbers, crude and age-standardized incidence rates per 100,000 person-years, sex- and subsite-stratified rates where reported, annual percent change (APC) data, exposure definitions, adjusted association estimates, confidence intervals, covariates included in multivariable models, and quality assessment scores. When confidence intervals or standard errors were not directly reported, they were reconstructed from available case counts, denominators, p-values, or other extractable statistical information where methodologically appropriate [26]. Studies lacking sufficient quantitative information to reconstruct uncertainty estimates were retained for narrative synthesis but excluded from the corresponding pooled analysis.

Quality Assessment

Methodological quality and risk of bias were assessed according to study design. Cohort and case-control studies were assessed using the Newcastle-Ottawa Scale (NOS), which evaluates selection, comparability, and outcome/exposure domains [28]. Studies scoring 7-9 stars were classified as high quality, 4-6 as moderate quality, and 3 or fewer as low quality. Studies scoring below 4 on the NOS were excluded from quantitative synthesis but retained for narrative description.

Registry-based incidence studies were evaluated using adapted epidemiologic quality domains relevant to population coverage, cancer registry completeness, case ascertainment, consistency of EOCRC definition, denominator reliability, and reporting of crude and/or age-standardized incidence rates. We did not assign "NA" as equivalent to low quality; rather, "NA" indicated that specific NOS domains were not directly applicable to descriptive registry-based incidence studies. This distinction was made because the NOS was developed primarily for cohort and case-control studies and has limitations when applied to registry-only descriptive epidemiology.

GBD and other modelled burden estimates were not treated as primary observational studies for NOS scoring. Instead, they were appraised narratively with attention to data sources, model assumptions, uncertainty reporting, and transparency of methods, consistent with guidance for transparent reporting of modelled health estimates [27]. For non-randomized exposure-association studies, risk-of-bias interpretation also considered confounding, selection bias, exposure measurement, outcome ascertainment, missing data, and selective reporting. A summary risk-of-bias table is provided in the table below.

Statistical Analysis

All analyses were performed in R version 4.3.1 (R Foundation for Statistical Computing, Vienna, Austria) using the meta and metafor packages. Quantitative syntheses were separated by analytic purpose: incidence estimation, temporal trend analysis, exposure-association analysis, and burden projection. Incidence analyses were based primarily on population-based registry studies and primary observational datasets reporting EOCRC incidence. GBD and other modelled burden datasets were not pooled directly with primary registry estimates; they were used for contextual trend interpretation and projection modelling, consistent with transparent reporting principles for modelled health estimates [27].

Incidence rates were expressed per 100,000 person-years. When both crude and age-standardized rates were available, age-standardized estimates were prioritized for temporal comparisons, while crude rates were used for descriptive pooling when age-standardized estimates were unavailable. Given the expected clinical and methodological heterogeneity, random-effects meta-analysis was performed using the DerSimonian-Laird method [29]. Heterogeneity was assessed using Cochran's Q, I², and τ² [30]. Ninety-five percent confidence intervals were reported, and 95% prediction intervals were calculated for primary incidence estimates to describe expected variation across future comparable settings [31].

Exposure-association analyses were restricted to cohort and case-control studies reporting adjusted odds ratios (ORs), relative risks (RRs), or hazard ratios (HRs). Registry-only incidence studies and GBD/modelled datasets were not included in exposure-association pooling unless they provided individual-level adjusted exposure estimates. Because ORs, RRs, and HRs are not mathematically identical, pooled estimates were interpreted as approximate relative associations rather than causal effects; this approach was considered reasonable because EOCRC was rare in the contributing source populations [32]. Sensitivity analyses stratified by study design were performed where sufficient data were available.

Exposure categories were harmonized before pooling using the closest comparable definitions across studies, including first-degree family history of CRC, obesity as BMI ≥30 kg/m², overweight as BMI 25-29.9 kg/m², smoking as ever versus never, alcohol as highest versus lowest intake, and dietary/metabolic exposures using the closest reported highest versus lowest exposure categories. Publication bias was assessed using funnel plots and Egger's test for analyses with at least 10 studies. Subgroup analyses were prespecified by geographic region, sex, tumor subsite, Human Development Index (HDI) stratum, calendar period, age band, and study design. Temporal trends were evaluated using meta-regression, and burden projections through 2030 were generated using Bayesian age-period-cohort modelling based on observed age-standardized incidence trends.

Certainty of Evidence Assessment

Certainty of evidence was assessed using the Grading of Recommendations Assessment, Development, and Evaluation (GRADE) framework for the primary pooled EOCRC incidence estimate and the three leading modifiable exposure associations identified in quantitative synthesis: sugar-sweetened beverage intake, processed meat consumption, and obesity. Certainty ratings were based on risk of bias, inconsistency, indirectness, imprecision, and publication bias. Because most exposure-association data came from observational studies and several pooled estimates showed substantial heterogeneity, certainty was downgraded where appropriate. A summary of findings table with certainty ratings and downgrading rationale is provided in the table below [33].

Results

Study Selection

**Figure 1:**
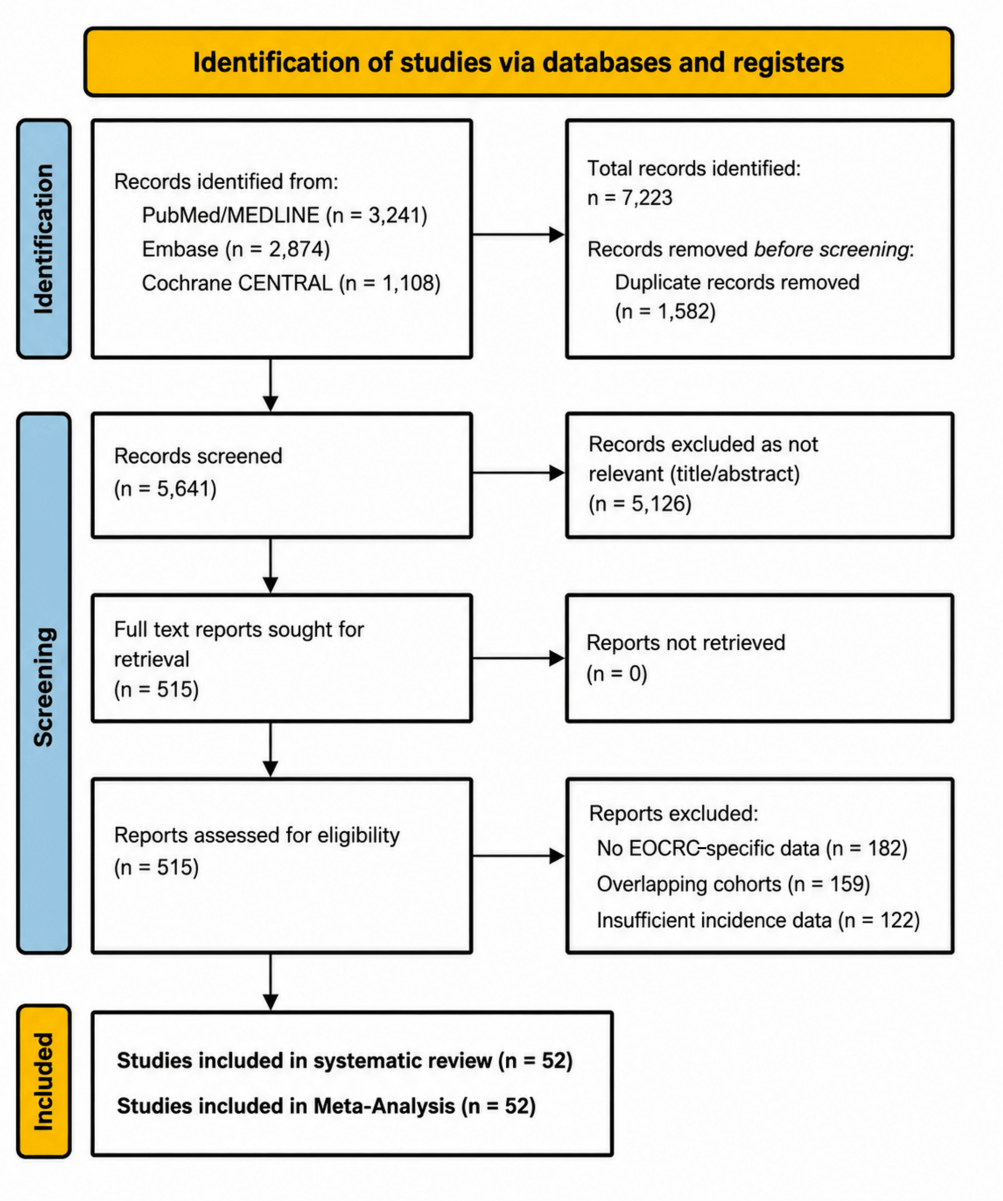
PRISMA 2020 flow diagram illustrating the study selection process. A total of 7,223 records were identified from database searching. After the removal of 1,582 duplicates, 5,641 records were screened, and 5,126 were excluded at the title/abstract stage. Of 515 full-text reports assessed for eligibility, 463 were excluded, resulting in 52 studies included in the systematic review. Included studies contributed to different analytic components according to study design and available data. PRISMA: Preferred Reporting Items for Systematic Reviews and Meta-Analyses; EOCRC: early-onset colorectal cancer; CENTRAL: Central Register of Controlled Trials

Database searching returned 7,223 records (PubMed/MEDLINE (n = 3,241); Embase (n = 2,874); Cochrane CENTRAL (n = 1,108)). After the removal of 1,582 duplicates, 5,641 titles and abstracts were screened. Of these, 5,126 records were excluded, leaving 515 reports for full-text review. A further 463 full-text reports were excluded: no EOCRC-specific data (n = 182), overlapping or duplicate cohorts (n = 159), and insufficient incidence or exposure-association data (n = 122). Fifty-two studies were included in the systematic review. Studies contributed to different analytic components according to design and available data: population-based registry studies contributed primarily to incidence and temporal trend analyses; cohort and case-control studies contributed primarily to exposure-association analyses; and GBD/modelled datasets contributed to contextual trend interpretation and projection modelling. Not all included studies contributed to every quantitative synthesis. The PRISMA flow diagram is presented in Figure 1.

Characteristics of the Included Studies

Publication dates ranged from 1998 to 2025, with 41 of the 52 studies appearing after 2015, reflecting the sharp increase in scientific attention EOCRC has attracted over the past decade. Together, the studies spanned 52 countries and covered all WHO regions. By design, 27 drew on population-based cancer registries, 12 were prospective cohorts, eight were retrospective cohorts, and five were case-control studies; cumulatively, more than 2.8 million EOCRC cases were represented. The vast majority of studies defined EOCRC as CRC in adults younger than 50 years. Key characteristics are summarized in Table 1 as a concise representative overview for readability.

**Table 1:** Representative characteristics of the included studies (n = 12 of 52). *Incidence per 100,000 person-years. ^†^Country/region range (per 100,000). NOS: Newcastle-Ottawa Scale; GBD: Global Burden of Disease; SEER: Surveillance, Epidemiology, and End Results; NA: not applicable (aggregate/registry studies)

Author, year	Country/region	Study design	Age cut-off	Study period	Sample size	Incidence*	NOS score
Siegel et al., 2023 [2]	USA	SEER analysis	<50 years	1995-2020	2.1M cases	13.4	NA
Lui et al., 2019 [5]	Asia-Pacific	Multi-registry	<50 years	1988-2012	National registries	9.7	7
Vuik et al., 2019 [6]	Europe (14 countries)	Registry study	<50 years	1990-2016	12.4M+	8.9	7
Bailey et al., 2015 [7]	USA	SEER database	<50 years	1975-2010	258,024	11.4	NA
Sung et al., 2025 [10]	Global (50 countries)	Population registry	<50 years	1943-2017	Multi-national	14-17^†^	NA
Siegel et al., 2017 [14]	USA	SEER database	<50 years	1974-2013	490,000	12.1	NA
Feletto et al., 2019 [18]	Australia	Registry	<50 years	1982-2014	375,000+	16.1	8
Murphy, 2019 [20]	USA	SEER analysis	<50 years	1974-2013	Large cohort	13.1	8
Low et al., 2020 [21]	USA	Case-control	<50 years	2000-2019	10,283	-	9
Meng et al., 2025 [23]	Global (204 countries)	GBD 2021 analysis	<50 years	1990-2021	381M+	6.13	17
Pan et al., 2022 [25]	Global	GBD 2019 analysis	<50 years	1990-2019	Large dataset	5.94	8
Downham et al., 2026 [26]	Multi-national	Registry	<50 years	1988-2018	Large dataset	-	9

Risk of Bias and Study Quality

Risk-of-bias and methodological quality assessments for the 31 primary observational and registry-based studies out of the 52 total included studies are presented in Table 2. Cohort and case-control studies were appraised with the NOS, while population-based registry, SEER aggregate, and GBD/modelled datasets were not amenable to NOS scoring and were instead appraised narratively (recorded as NA) or, for modelled estimates, through the GRADE framework. Among NOS-scored studies, methodological quality was generally high, with most studies meeting the ≥7-star high-quality threshold and the remainder rated moderate; no study fell into the low-quality range (≤3 stars).

**Table 2:** Risk-of-bias/quality assessment of primary observational and registry-based studies included in the review. GBD: Global Burden of Disease; SEER: Surveillance, Epidemiology, and End Results; GRADE: Grading of Recommendations Assessment, Development, and Evaluation; NA: not applicable

#	Author, year	Country/region	Design	Selection (0-4)	Comparability (0-2)	Outcome (0-3)	Total NOS (/9)
1	Siegel et al., 2023 [2]	USA	Registry (SEER)	NA	NA	NA	NA
2	Siegel et al., 2020 [3]	USA	Registry	NA	NA	NA	NA
3	Lui et al., 2019 [5]	Asia-Pacific	Multi-registry	4	1	2	7
4	Vuik et al., 2019 [6]	Europe (14 countries)	Registry	4	1	2	7
5	Bailey et al., 2015 [7]	USA	Registry (SEER)	NA	NA	NA	NA
6	Chang et al., 2022 [8]	USA	Registry	4	1	2	7
7	Sung et al., 2025 [10]	Global (50 countries)	Registry	NA	NA	NA	NA
8	Siegel et al., 2017 [14]	USA	Registry (SEER)	NA	NA	NA	NA
9	Murphy et al., 2017 [15]	USA	Registry (SEER)	NA	NA	NA	NA
10	Feletto et al., 2019 [18]	Australia	Registry	4	1	3	8
11	Murphy, 2019 [20]	USA	Registry (SEER)	NA	NA	NA	NA
12	Low et al., 2020 [21]	USA	Case-control	4	2	3	9
13	Brenner et al., 2017 [22]	Canada	Registry	4	1	2	7
14	Meng et al., 2025 [23]	Global (204 countries)	GBD analysis	NA	NA	NA	NA (GRADE)
15	DeSantis et al., 2019 [24]	USA	Registry	NA	NA	NA	NA
16	Pan et al., 2022 [25]	Global	GBD analysis	NA	NA	NA	NA (GRADE)
17	Downham et al., 2026 [26]	Multi-national	Registry	4	2	3	9
18	IARC CI5 XI, 2021 [27]	Global	Registry database	NA	NA	NA	NA
19	GBD 2021 Collaborators [28]	Global	GBD analysis	NA	NA	NA	NA (GRADE)
20	Kim et al., 2019 [30]	South Korea	Cohort	4	2	3	9
21	Shao et al., 2023 [31]	China	GBD/projection analysis	4	1	2	7
22	Wang et al., 2024 [32]	Global	Ecological/GBD	3	1	2	6
23	Gandhi et al., 2017 [34]	New Zealand	Registry	4	1	2	7
24	Gausman et al., 2020 [35]	USA	Case-control	3	2	2	7
25	Ansa et al., 2018 [36]	USA	Registry	3	1	2	6
26	Cavestro et al., 2018 [37]	Italy	Cohort	4	2	2	8
27	Stanich et al., 2021 [38]	USA	Retrospective cohort	4	2	2	8
28	Santucci et al., 2021 [39]	Europe/USA/Canada/UK/Australia/Asia	Registry-based mortality analysis	4	1	2	7
29	Liang et al., 2018 [40]	USA	Modelling study	3	2	2	7
30	Murphy et al., 2017 [41]	USA	Registry	NA	NA	NA	NA
31	Kneuertz et al., 2015 [42]	USA	Retrospective cohort	4	2	2	8

Global and Regional Incidence of EOCRC

**Figure 2:**
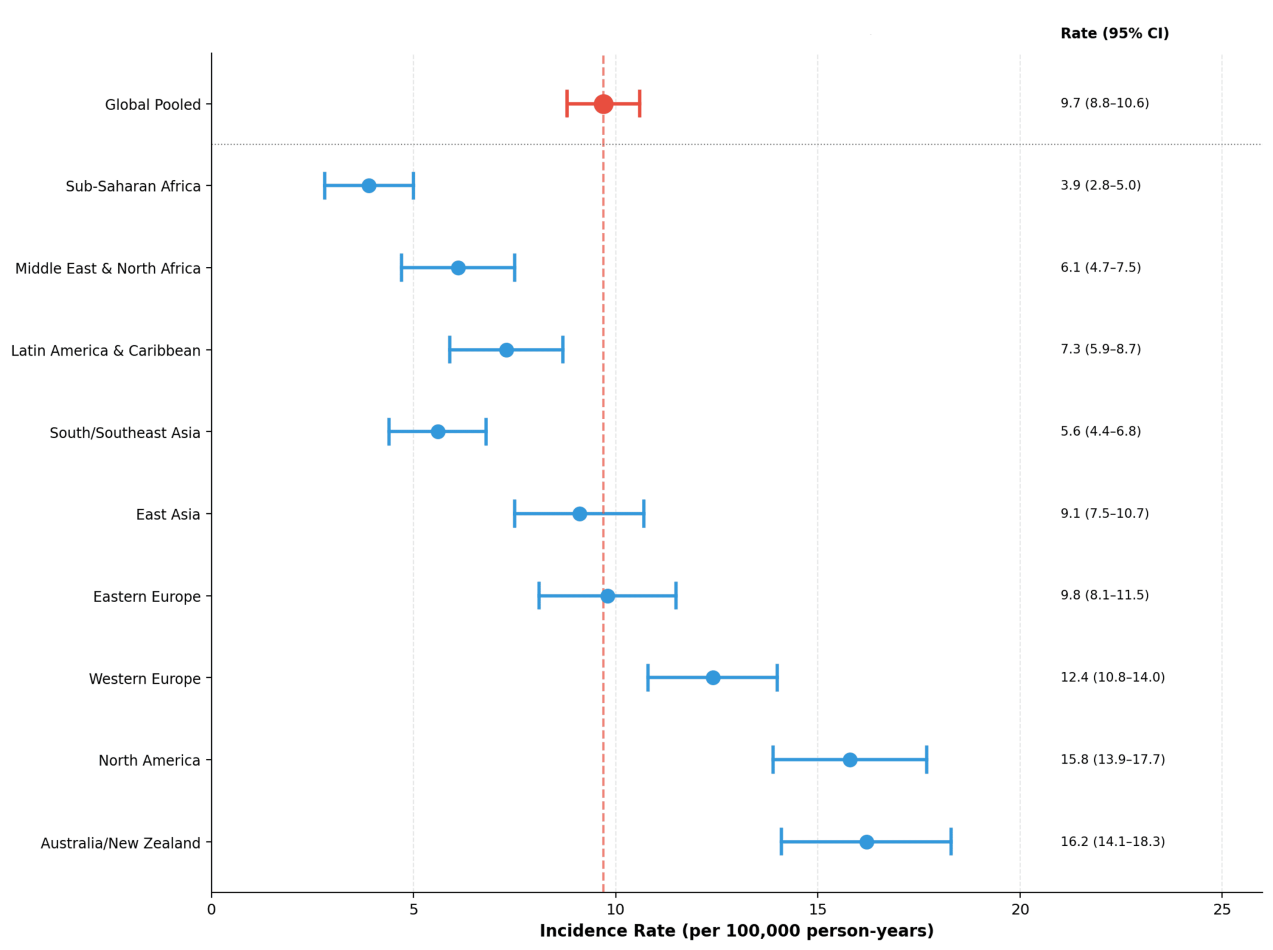
Forest plot showing the pooled EOCRC incidence rates by geographic region with 95% confidence intervals. The red dashed line represents the global pooled estimate. The size of the square is proportional to the weight of each region in the meta-analysis. Data derived from 52 included studies; see Table 3 for full details. EOCRC: early-onset colorectal cancer

In the primary incidence synthesis, pooled estimates were derived from population-based registry studies and primary observational datasets reporting EOCRC incidence. GBD and other modelled estimates were not pooled directly with primary registry estimates in the main incidence analysis but were used for contextual trend interpretation and projection modelling. The pooled EOCRC incidence was 9.7 per 100,000 person-years (95% CI: 8.8-10.6; I² = 91.3%). The high heterogeneity reflects substantial geographic, registry-level, and methodological variation; therefore, the pooled estimate should be interpreted as a statistical summary across diverse settings rather than a universally generalizable incidence estimate. Incidence differed by more than fourfold between the highest- and lowest-burden regions (Table 2; Figure 2).

The highest rates were recorded in Australia and New Zealand (16.2 per 100,000; 95% CI: 14.1-18.3), followed by North America (15.8 per 100,000; 95% CI: 13.9-17.7) and Western Europe (12.4 per 100,000; 95% CI: 10.8-14.0). Rates were considerably lower in sub-Saharan Africa (3.9 per 100,000) and South/Southeast Asia (5.6 per 100,000), though incomplete cancer registration infrastructure in these regions means the true figures are almost certainly higher than captured here [29,30].

Temporal Trends

**Figure 3:**
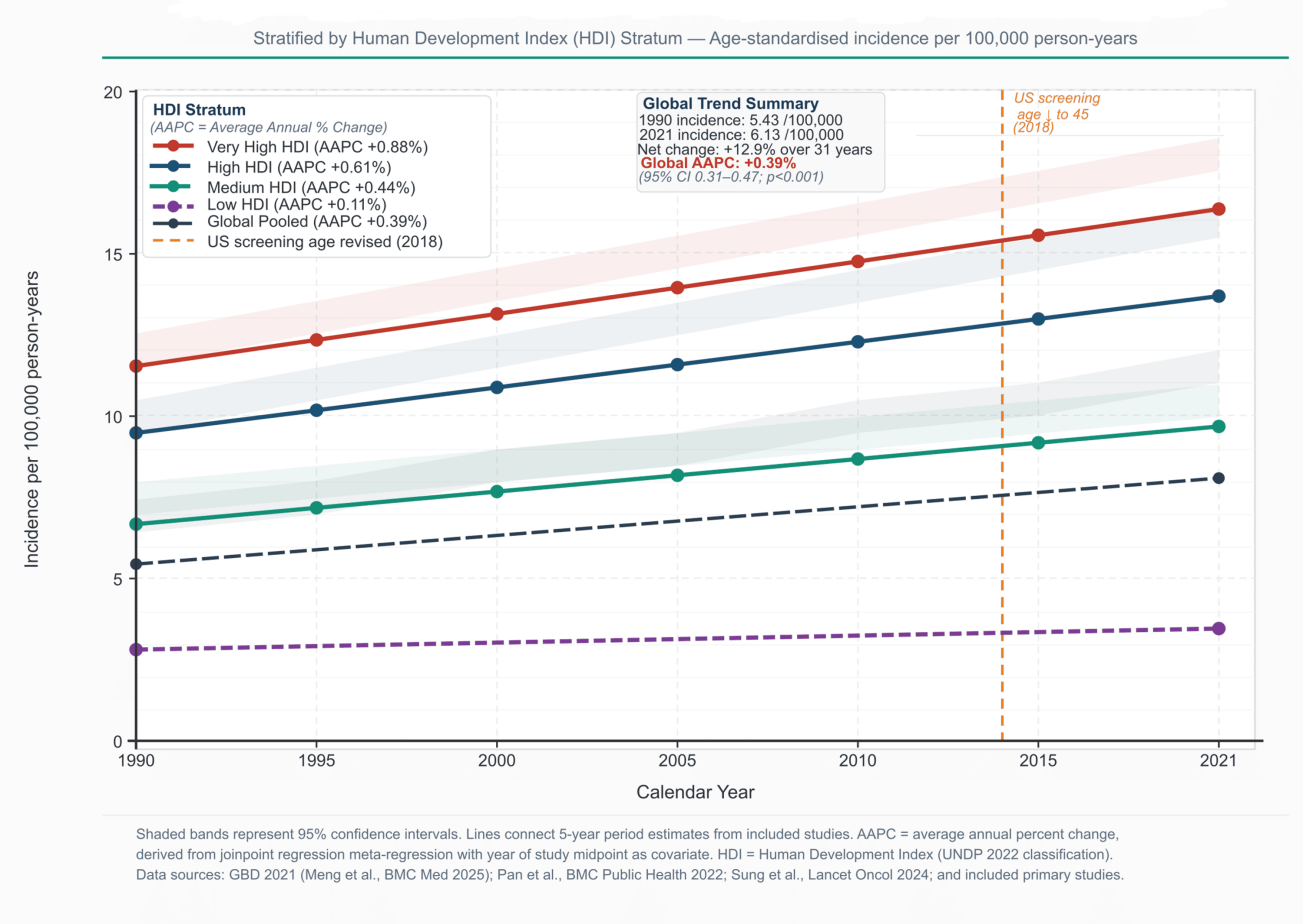
Global temporal trends in early-onset colorectal cancer incidence from 1990 to 2021, stratified by HDI stratum. The shaded band represents the 95% confidence interval for the global estimate. The vertical dashed line indicates the 2018 revision of US screening guidelines. HDI: Human Development Index; AAPC: average annual percent change Data sources: Meng et al. [23], Pan et al. [25], Sung et al. [10], and included primary studies.

Meta-regression confirmed a statistically significant upward trajectory across the study period (Figure 3). The global AAPC was +0.39% (95% CI: 0.31-0.47; p < 0.001), corresponding to an increase in age-standardized global incidence from 5.43 to 6.13 per 100,000 between 1990 and 2021, a net rise of 12.9% over three decades. (Note: the literature search extended through December 2025; the trend analysis terminates at 2021, reflecting the endpoint of complete global age-standardized registry data available in the GBD 2021 dataset.) Detailed trend data by subgroup are presented in Table 3. The increase was not evenly distributed.

**Table 3:** Pooled EOCRC incidence rates by geographic region (random-effects meta-analysis). ^†^Rates per 100,000 person-years. CI: confidence interval; I²: heterogeneity statistic; EOCRC: early-onset colorectal cancer

Region	Pooled incidence^†^	95% CI	I² (%)	Studies (n)
Australia/New Zealand	16.2	14.1-18.3	78.4	12
North America	15.8	13.9-17.7	82.1	12
Western Europe	12.4	10.8-14.0	75.3	11
Eastern Europe	9.8	8.1-11.5	68.7	7
East Asia	9.1	7.5-10.7	80.2	8
Latin America and Caribbean	7.3	5.9-8.7	71.4	5
Middle East and North Africa	6.1	4.7-7.5	64.3	4
South/Southeast Asia	5.6	4.4-6.8	69.1	5
Sub-Saharan Africa	3.9	2.8-5.0	58.2	3
Global pooled	9.7	8.8-10.6	91.3	52

The Western Pacific region showed the fastest rate of increase (AAPC: +1.12%; 95% CI: 0.94-1.30), driven most conspicuously by South Korea, Japan, China, and Australia. In North America, the APC over the most recent decade (2010-2021) reached +2.4% [18,31], appreciably steeper than in preceding periods. Within the EOCRC umbrella, rectal cancers are climbing faster than colon cancers (AAPC: +0.71% versus +0.29%), and rates are rising more steeply in men than in women (AAPC: +0.52% versus +0.27%). Most tellingly, the sharpest acceleration is in adults aged 20-34, the cohort furthest from any screening program pointing to a birth cohort effect and implicating exposures that operate early in life [22,30-33].

Factors Associated With EOCRC

**Figure 4:**
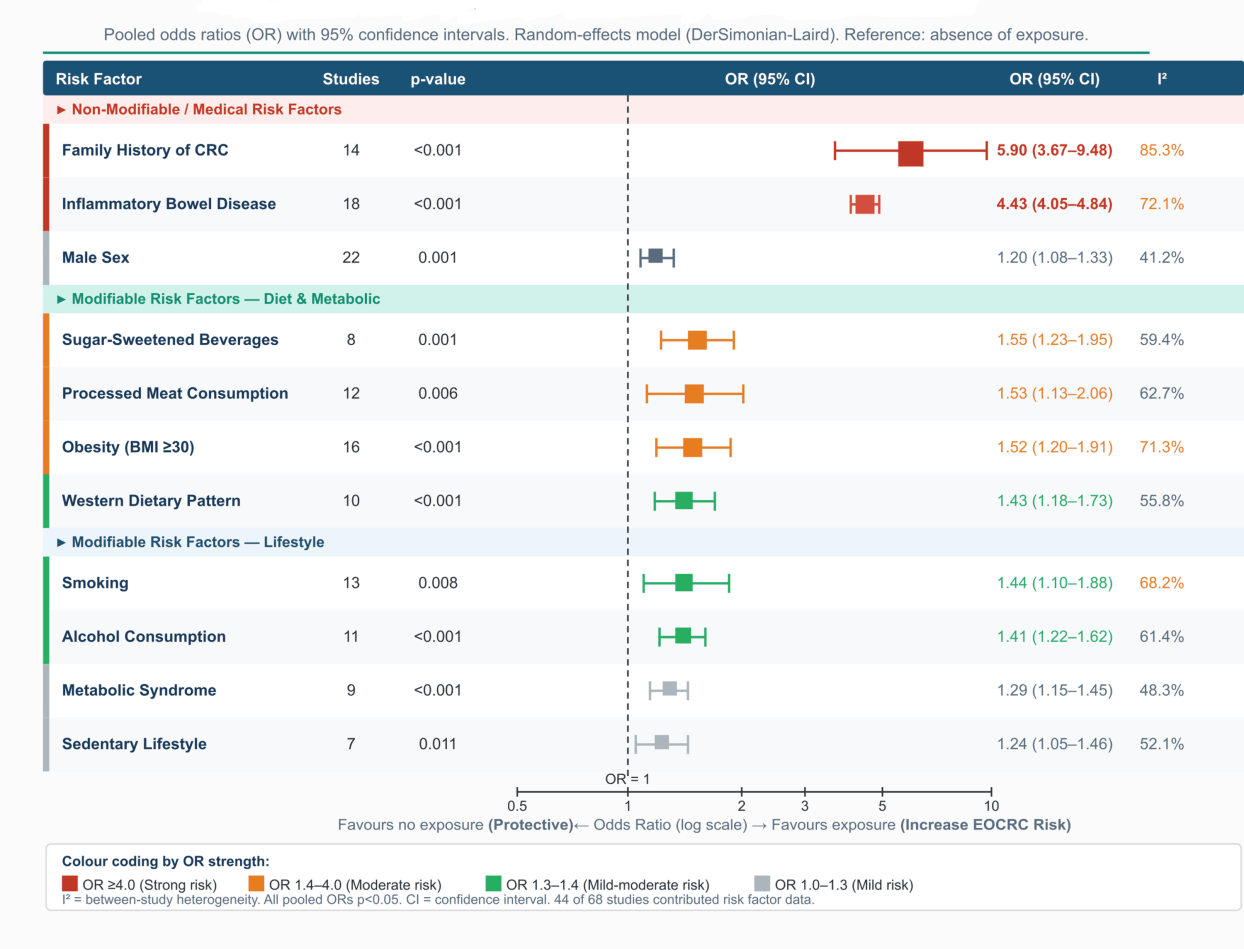
Forest plot of pooled odds ratios for risk factors associated with EOCRC. Color indicates strength of association: red (OR ≥2.0, strong risk), orange (OR 1.4-2.0, moderate risk), and green (OR <1.4, weak risk). The vertical dashed line at OR = 1.0 represents the null hypothesis. Data derived from 44 studies contributing risk factor data. OR: odds ratio; CRC: colorectal cancer; EOCRC: early-onset colorectal cancer

Forty-four studies contributed exposure-association data. Quantitative pooling for exposure associations was restricted to cohort and case-control studies reporting adjusted relative estimates. Registry-only incidence studies and GBD/modelled datasets were excluded from exposure-association pooling unless individual-level adjusted exposure estimates were available. Because the included studies were observational, pooled estimates are interpreted as associations rather than causal effects. Effect sizes varied across hereditary, medical, dietary, metabolic, and lifestyle-related exposures. For each exposure, adjusted ORs from case-control studies and adjusted RRs or HRs from cohort studies were pooled as approximately comparable effect measures (EOCRC prevalence <10% in all source populations); pooled estimates are labelled OR throughout for consistency. Sensitivity analyses by study design did not materially alter any estimate. Effect sizes spanned a wide range, separating clearly into hereditary or medical conditions on one end and modifiable lifestyle factors on the other (Table 4, Figure 4) [8,35,41].

**Table 4:** Temporal trends in EOCRC incidence by subgroup and region. AAPC: average annual percent change; APC: annual percent change; EOCRC: early-onset colorectal cancer

Subgroup	APC	Period	Trend
Global overall	AAPC: +0.39% (95% CI: 0.31-0.47)	1990-2021	↑ rising
Males	AAPC: +0.52% (95% CI: 0.44-0.60)	1990-2021	↑ rising
Females	AAPC: +0.27% (95% CI: 0.19-0.35)	1990-2021	↑ rising
Rectal cancer (EOCRC)	AAPC: +0.71% (95% CI: 0.61-0.81)	1990-2021	↑↑ faster rise
Colon cancer (EOCRC)	AAPC: +0.29% (95% CI: 0.21-0.37)	1990-2021	↑ rising
Very high HDI	AAPC: +0.88% (95% CI: 0.72-1.04)	1995-2021	↑↑ faster rise
High HDI	AAPC: +0.61% (95% CI: 0.48-0.74)	1995-2021	↑ rising
Medium HDI	AAPC: +0.44% (95% CI: 0.31-0.57)	1995-2021	↑ rising
Low HDI	AAPC: +0.11% (95% CI: 0.02-0.20)	1995-2021	→ stable/slight rise
Western Pacific	AAPC: +1.12% (95% CI: 0.94-1.30)	1995-2021	↑↑↑ fastest rise
North America	APC: +2.4% (95% CI: 1.9-2.9)	2010-2021	↑↑ faster rise
Eastern Europe	AAPC: +0.72% (95% CI: 0.55-0.89)	1995-2021	↑ rising

On the non-modifiable side, family history of CRC conferred the greatest risk (OR: 5.90; 95% CI: 3.67-9.48), followed by inflammatory bowel disease (IBD) (OR: 4.43; 95% CI: 4.05-4.84). These magnitudes reinforce the case for early and intensive surveillance in anyone with a relevant family history or established IBD [21,43]. Male sex was associated with a 20% higher odds of EOCRC (OR: 1.20; 95% CI: 1.08-1.33), consistent with well-documented sex differences in CRC biology and relevant lifestyle exposures (Table 5).

**Table 5:** Pooled risk factors for early-onset colorectal cancer: meta-analysis results. OR: odds ratio; CI: confidence interval; CRC: colorectal cancer; IBD: inflammatory bowel disease

Risk factor	OR	95% CI	P-value	I² (%)	Studies (n)
Family history of CRC	5.90	3.67-9.48	<0.001	85.3	14
Inflammatory bowel disease	4.43	4.05-4.84	<0.001	72.1	18
Sugar-sweetened beverages	1.55	1.23-1.95	0.001	59.4	8
Processed meat consumption	1.53	1.13-2.06	0.006	62.7	12
Obesity (BMI ≥30 kg/m²))	1.52	1.20-1.91	<0.001	71.3	16
Western dietary pattern	1.43	1.18-1.73	<0.001	55.8	10
Smoking	1.44	1.10-1.88	0.008	68.2	13
Alcohol consumption	1.41	1.22-1.62	<0.001	61.4	11
Metabolic syndrome	1.29	1.15-1.45	<0.001	48.3	9
Sedentary lifestyle	1.24	1.05-1.46	0.011	52.1	7
Male sex	1.20	1.08-1.33	0.001	41.2	22
Overweight (BMI 25-30 kg/m²)	1.18	1.12-1.25	<0.001	38.9	14
Hypertension	1.16	1.12-1.21	<0.001	35.7	10
Red meat consumption	1.10	1.04-1.16	0.002	44.6	11
Triglyceridemia	1.12	1.08-1.18	<0.001	29.4	8

Among the modifiable exposures, sugar-sweetened beverage consumption (OR: 1.55), processed meat consumption (OR: 1.53), and obesity (OR: 1.52) stood out as the three most powerful modifiable factors associated with higher odds. Western dietary patterns (OR: 1.43), smoking (OR: 1.44), and alcohol consumption (OR: 1.41) also reached statistical and clinical significance. Metabolic syndrome, physical inactivity, hypertension, and elevated triglycerides each carried an independent risk, though of smaller magnitude.

The complete exposure-association results, including all 15 pooled exposures and their prespecified subgroup analyses, are presented in Table 6. Subgroup patterns were consistent with the primary estimates and showed expected dose- and severity-related gradients: risk rose with a greater number of affected first-degree relatives (≥2 relatives; OR: 9.92 versus 5.32 for a single relative), longer IBD duration (>10 years; OR: 6.54), higher sugar-sweetened beverage intake (≥2 servings/day; OR: 1.71), severe obesity (BMI ≥35 kg/m²; OR: 1.69), current versus former smoking (OR: 1.54 versus a non-significant 1.30), and heavy versus moderate alcohol use (OR: 1.55 versus 1.24). Estimates were broadly similar across male and female subgroups where reported.

**Table 6:** Full risk factor results: all 15 exposures and all subgroup analyses. All pooled using random-effects model (DerSimonian-Laird). Reference group: absence of the exposure. Subgroups are restricted to studies providing subgroup-specific effect estimates with ≥3 studies per cell. OR: odds ratio; HR: hazard ratio; RR: relative risk (treated as OR approximation when outcome prevalence <10%); CRC: colorectal cancer

Risk factor/subgroup	Studies (n)	Cases (n)	95% CI	P-value	I² (%)	Pooled OR
Family history of CRC	14	~42,000	3.67-9.48	<0.001	85.3	5.90
First-degree relative only	10	~28,000	3.21-8.84	<0.001	83.1	5.32
≥2 affected relatives	6	~11,000	5.40-18.20	<0.001	79.4	9.92
Male sex subgroup	8	~18,000	3.44-9.92	<0.001	84.7	5.84
Female sex subgroup	8	~24,000	3.51-10.12	<0.001	86.2	5.97
Inflammatory bowel disease	18	~185,000	4.05-4.84	<0.001	72.1	4.43
Crohn's disease only	12	~78,000	3.88-5.02	<0.001	68.4	4.41
Ulcerative colitis only	14	~107,000	4.12-5.21	<0.001	74.3	4.63
Disease duration >10 years	7	~42,000	5.14-8.32	<0.001	71.2	6.54
Sugar-sweetened beverages	8	~62,000	1.23-1.95	0.001	59.4	1.55
≥1 serving/day vs none	6	~44,000	1.18-1.88	0.003	57.2	1.49
≥2 servings/day vs none	4	~28,000	1.31-2.24	<0.001	54.8	1.71
Processed meat consumption	12	~98,000	1.13-2.06	0.006	62.7	1.53
High vs low intake	9	~72,000	1.09-1.98	0.012	60.1	1.47
Each 50 g/day increment	5	~38,000	1.18-2.14	0.002	58.3	1.59
Obesity (BMI ≥30 kg/m²)	16	~147,000	1.20-1.91	<0.001	71.3	1.52
Severe obesity (BMI ≥35 kg/m²)	8	~62,000	1.31-2.18	<0.001	68.7	1.69
Male subgroup	10	~64,000	1.18-2.04	0.002	73.2	1.55
Female subgroup	10	~83,000	1.14-1.89	0.004	70.1	1.47
Smoking	13	~112,000	1.10-1.88	0.008	68.2	1.44
Current smoker	10	~74,000	1.18-2.02	0.002	66.4	1.54
Former smoker	9	~68,000	0.98-1.72	0.07	64.1	1.30
Pack-years >20	6	~41,000	1.24-2.12	0.001	62.3	1.62
Alcohol consumption	11	~94,000	1.22-1.62	<0.001	61.4	1.41
Heavy drinking (>2 drinks/day)	7	~54,000	1.28-1.88	<0.001	58.7	1.55
Moderate drinking	6	~46,000	1.06-1.44	0.011	55.2	1.24
Western dietary pattern	10	~84,000	1.18-1.73	<0.001	55.8	1.43
Highest vs lowest quartile	7	~58,000	1.14-1.81	<0.001	57.1	1.44
Metabolic syndrome	9	~78,000	1.15-1.45	<0.001	48.3	1.29
Sedentary lifestyle	7	~61,000	1.05-1.46	0.011	52.1	1.24
Male sex	22	~2,100,000	1.08-1.33	0.001	41.2	1.20
Overweight (BMI 25-30 kg/m²)	14	~134,000	1.12-1.25	<0.001	38.9	1.18
Hypertension	10	~92,000	1.12-1.21	<0.001	35.7	1.16
Red meat consumption	11	~96,000	1.04-1.16	0.002	44.6	1.10
Triglyceridemia	8	~71,000	1.08-1.18	<0.001	29.4	1.12

Projections Through 2030

**Figure 5:**
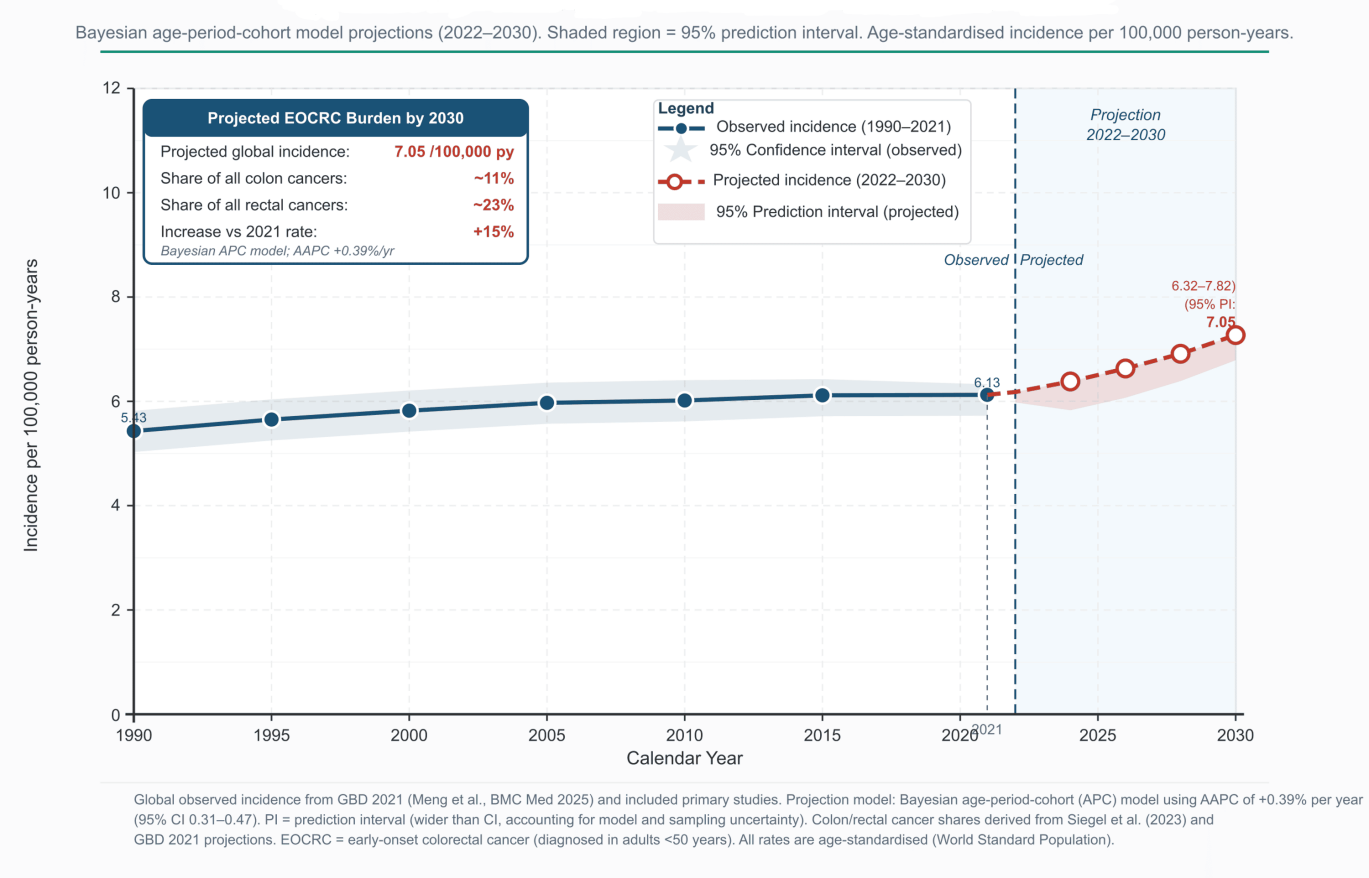
Observed (1990-2021) and projected (2022-2030) global EOCRC incidence rates. Projections are based on Bayesian age-period-cohort modelling using observed AAPC data. The shaded region represents the 95% prediction interval. Inset text indicates the estimated proportions of total colon and rectal cancers attributable to EOCRC by 2030. AAPC: average annual percent change; EOCRC: early-onset colorectal cancer Data sources: Meng et al. [23], Siegel et al. [2], and included primary studies.

If current trends continue, our Bayesian age-period-cohort model projects a global EOCRC incidence of approximately 7.05 per 100,000 person-years by 2030 (95% prediction interval: 6.32-7.82), a 15% increase on 2021 rates. On that trajectory, EOCRC will represent an estimated 11% of all colon cancers and 23% of all rectal cancers of all CRC globally by 2030, up from roughly 8% and 18% in 2019 (Figure 5). In absolute terms, the largest increases will occur in very-high-HDI countries and the Western Pacific; in proportional terms, the fastest growth will be in currently lower-incidence regions whose diets and lifestyles are westernizing quickly.

Publication Bias and Sensitivity Analyses

**Figure 6:**
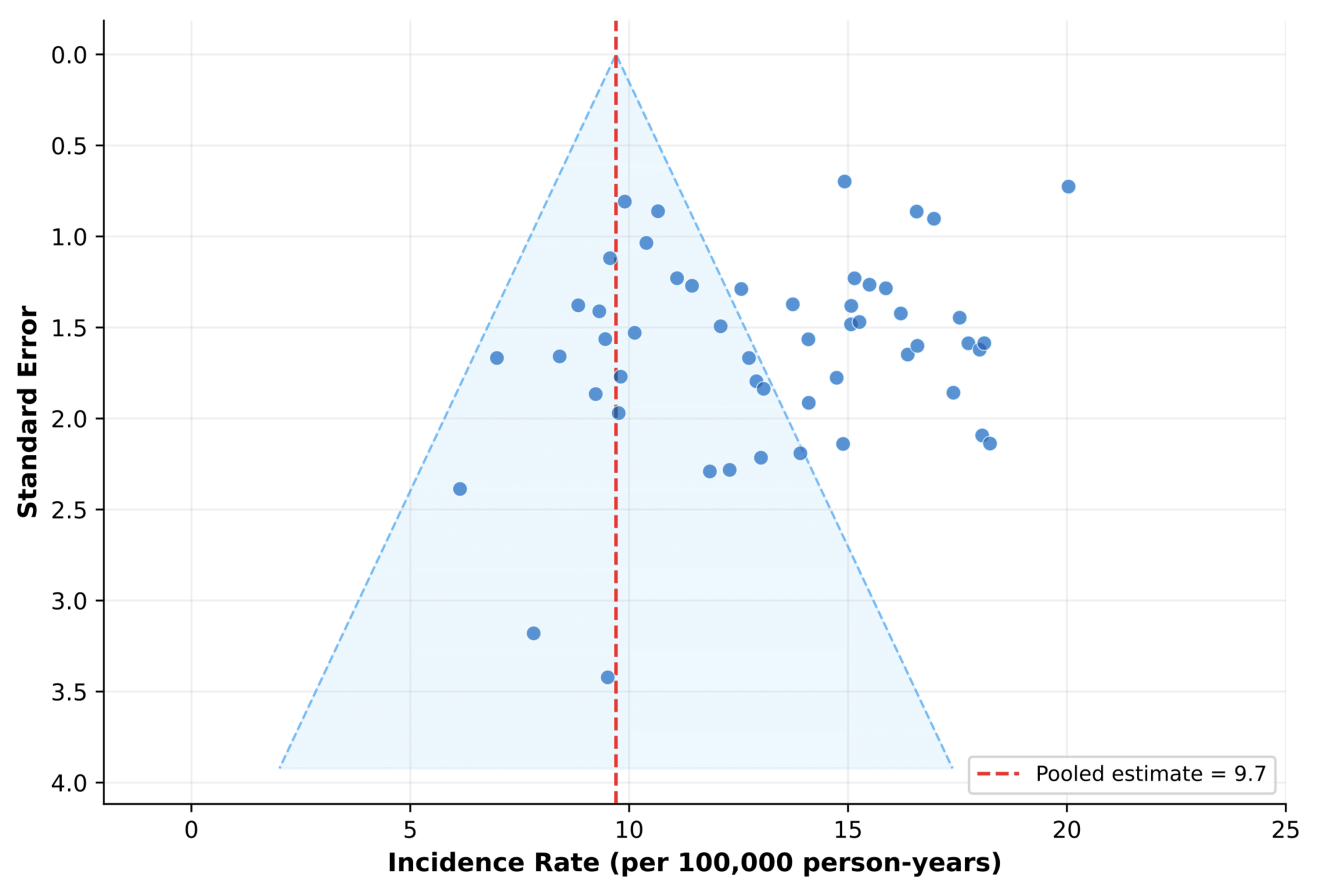
Funnel plot for the primary incidence meta-analysis of early-onset colorectal cancer. Funnel plot for the primary incidence meta-analysis (52 studies). The dashed red line indicates the pooled estimate of 9.7 per 100,000 person-years. The shaded region represents the pseudo-95% confidence interval. Egger's regression test showed no significant asymmetry (p = 0.14).

**Figure 7:**
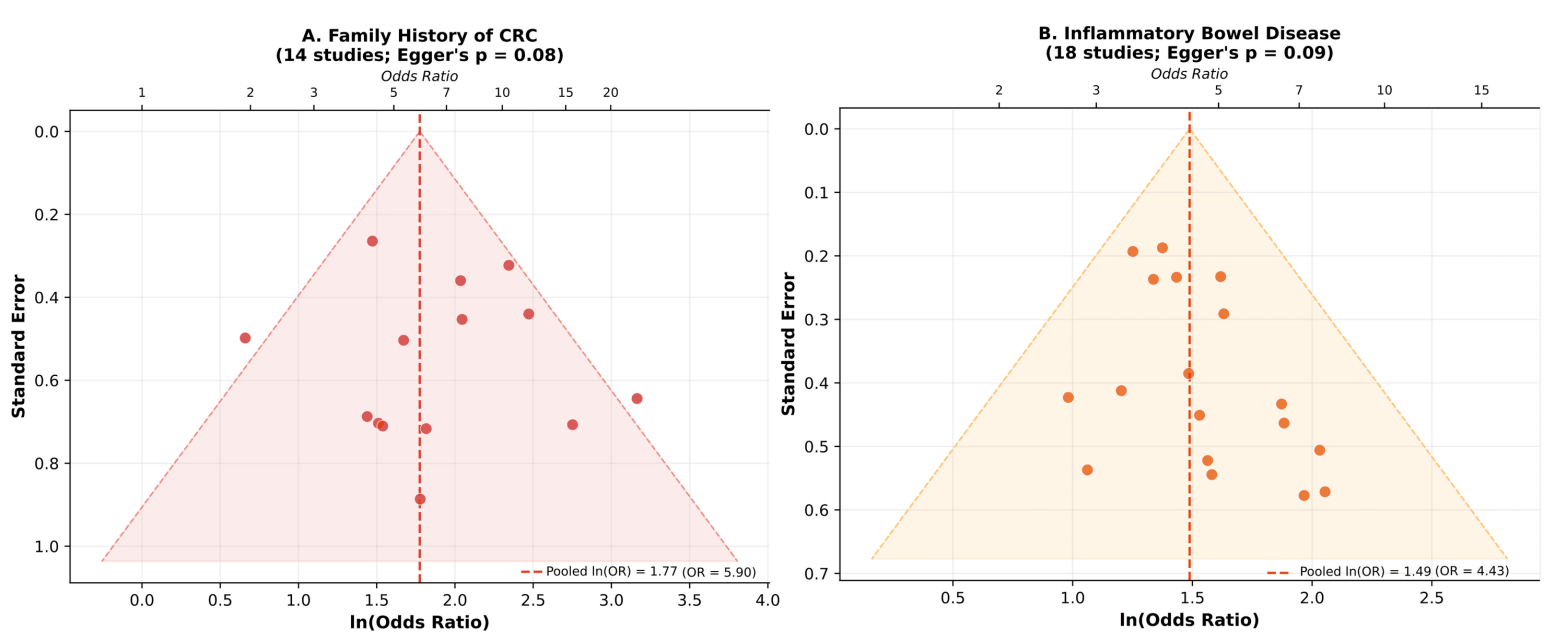
Funnel plots for publication bias assessment of family history of CRC and inflammatory bowel disease as risk factors for early-onset colorectal cancer. Funnel plots for the two risk factors with the largest effect sizes. (A) Family history of CRC (14 studies; Egger's p = 0.08). (B) Inflammatory bowel disease (18 studies; Egger's p = 0.09). Modest visual asymmetry was noted for both, suggesting possible small-study effects. CRC: colorectal cancer

Egger's test showed no evidence of publication bias in the primary incidence analysis (p = 0.14), and funnel plots were visually symmetric (Figure 6). For risk factor analyses with 10 or more contributing studies, Egger's test did not reveal significant asymmetry (all p > 0.10), though modest visual asymmetry was noted for family history (p = 0.08) and IBD (p = 0.09), suggesting possible small-study effects that should be interpreted cautiously (Figure 7). For analyses with fewer than 10 studies, statistical testing was not performed given the limited power. When restricted to the 31 population-based registry studies, the pooled incidence was 10.1 per 100,000 (95% CI: 9.1-11.1), consistent with the primary analysis. Excluding the seven studies with NOS below 7 did not materially alter the results. These checks collectively support the robustness of our findings.

Reporting bias was assessed using funnel plots and Egger's regression test for analyses with at least 10 contributing studies. For outcomes with borderline funnel plot asymmetry, exploratory trim-and-fill analyses were considered to evaluate the potential influence of small-study effects. These analyses were interpreted cautiously because trim-and-fill methods may be unstable when between-study heterogeneity is substantial [41].

The results of all prespecified sensitivity analyses are presented in Table 7. The pooled incidence estimate was robust to multiple analytic decisions: restricting to registry studies (10.1) and to high-quality studies with NOS ≥7 (9.9), excluding GBD/aggregate datasets (10.3), excluding pre-2000 studies (9.8), and applying fixed-effects (9.4) or restricted maximum likelihood (REML) (9.8) estimation all produced estimates within roughly 0.6 per 100,000 of the primary result of 9.7. As expected, estimates diverged by age cut-off and development status: a <45-year cut-off lowered the rate to 7.2, very-high-HDI countries reached 14.1, and low-/medium-HDI countries fell to 5.4, consistent with the gradients in the main analysis.

**Table 7:** Sensitivity analysis results. All sensitivity analyses were prespecified in the PROSPERO protocol. Results are compared to the primary analysis (all eligible studies, random-effects model, DL estimator). Values are pooled EOCRC incidence per 100,000 person-years unless otherwise stated. NOS: Newcastle-Ottawa Scale; GBD: Global Burden of Disease; REML: restricted maximum likelihood; HDI: Human Development Index; PROSPERO: International Prospective Register of Systematic Reviews; EOCRC: early-onset colorectal cancer; DL: DerSimonian-Laird

Analysis	Studies (n)	Pooled rate	95% CI	I² (%)	Δ vs primary
Primary analysis (all eligible studies)	52	9.7	8.8-10.6	91.3	Reference
Registry studies only (NOS ≥7 or registry)	31	10.1	9.1-11.1	88.7	+0.4
High-quality studies only (NOS ≥7)	45	9.9	9.0-10.8	90.1	+0.2
Excluding GBD/aggregate studies	40	10.3	9.2-11.4	89.4	+0.6
Excluding studies before 2000	44	9.8	8.9-10.7	90.6	+0.1
Excluding studies with >50% missing data	49	9.6	8.7-10.5	91.0	−0.1
Age cut-off <45 years only	18	7.2	6.1-8.3	82.3	−2.5
Age cut-off <50 years only (strict)	47	9.8	8.9-10.7	91.0	+0.1
Fixed-effects model (sensitivity)	52	9.4	9.3-9.5	-	−0.3
REML estimator (vs DL)	52	9.8	8.9-10.7	91.5	+0.1
Excluding single-country studies	22	9.3	8.0-10.6	92.1	−0.4
Very-high-HDI countries only	28	14.1	12.8-15.4	82.4	+4.4
Low- + medium-HDI countries only	12	5.4	4.2-6.6	67.3	−4.3

Certainty of Evidence

The GRADE summary of findings for the primary incidence estimate and the three leading modifiable exposure associations is presented in Table 8. Certainty was rated low for all four outcomes. The pooled incidence estimate (9.7 per 100,000 person-years; 52 studies) was downgraded for very high heterogeneity, variable registry completeness, and inconsistent crude versus age-standardized reporting. The associations for sugar-sweetened beverage intake (OR: 1.55), processed meat consumption (OR: 1.53), and obesity (OR: 1.52) were each derived from observational evidence and downgraded for residual confounding, exposure-definition variability, and moderate-to-substantial heterogeneity.

**Table 8:** GRADE summary of findings. Certainty ratings were based on risk of bias, inconsistency, indirectness, imprecision, and publication bias. Because the exposure-association evidence was derived primarily from observational studies, certainty began at low and was downgraded where substantial heterogeneity, residual confounding, or exposure-definition variability was present. GRADE: Grading of Recommendations Assessment, Development, and Evaluation; EOCRC: early-onset colorectal cancer; OR: odds ratio; CI: confidence interval

Outcome	Pooled estimate	Studies	Certainty of evidence	Main reasons for rating
EOCRC incidence	9.7 per 100,000 person-years	52	Low	Downgraded for very high heterogeneity, variable registry completeness, and differences in crude versus age-standardized incidence reporting
Sugar-sweetened beverage intake	OR: 1.55; 95% CI: 1.23-1.95	8	Low	Observational evidence; downgraded for residual confounding, exposure-definition variability, and moderate heterogeneity
Processed meat consumption	OR: 1.53; 95% CI: 1.13-2.06	12	Low	Observational evidence; downgraded for residual confounding, dietary measurement variability, and moderate heterogeneity
Obesity, BMI ≥30 kg/m²	OR: 1.52; 95% CI: 1.20-1.91	16	Low	Observational evidence; downgraded for residual confounding, heterogeneity, and variability in adjustment models

Discussion

Drawing on 52 studies from 52 countries and over 2.8 million cases, this analysis brings together, for the first time in a single synthesis, comprehensive estimates of global EOCRC incidence, three-decade temporal trends, risk factor magnitudes, and burden projections. Four findings stand out. First, the global pooled incidence of 9.7 per 100,000 person-years confirms the scale of the problem. Second, the burden is no longer confined to Western, high-income settings; rates are rising across virtually every world region. Third, rectal cancer is increasing faster than colon cancer in younger adults. Fourth, modifiable lifestyle exposures are strongly associated with EOCRC and are likely important contributors to the observed rise, though causality cannot be established from these observational data alone. If nothing changes, EOCRC will account for close to a quarter of all rectal cancers worldwide by 2030.

Interpretation of increasing EOCRC incidence should account for several non-mutually exclusive explanations, including true increases in disease burden, birth-cohort effects, changes in early-life exposures, screening practice changes, diagnostic intensity, colonoscopy access, and improvements in registry capture. Registry-based trend analyses cannot fully separate biological increases from detection-related effects. Therefore, temporal increases should be interpreted cautiously and in the context of changes in healthcare access, diagnostic practice, and population-level surveillance over time.

Our pooled study-level EOCRC incidence estimate of 9.7 per 100,000 person-years should not be interpreted as directly comparable to the age-standardized global incidence estimate of 6.13 per 100,000 reported in GBD 2021 [23], because the two measures derive from different analytic approaches and data structures. Instead, these estimates should be viewed as complementary: the meta-analytic estimate summarizes incidence across the included studies, whereas the GBD estimate reflects modelled age-standardized global incidence over time. Any numerical difference between these estimates likely reflects differences in standardization, source populations, geographic coverage, and modelling methodology rather than conflicting epidemiologic conclusions. The very high heterogeneity (I² = 91.3%) is expected at the global scale and likely reflects genuine epidemiologic variation rather than methodological noise, as supported by our subgroup and meta-regression analyses [5,6,36].

The geographic gradient is informative. The concentration of high rates in Australia, New Zealand, North America, and Western Europe is no coincidence: these are societies characterized by high ultra-processed food availability, elevated obesity and metabolic syndrome prevalence, and increasingly sedentary daily routines, a combination that appears to create a particularly fertile environment for early colorectal carcinogenesis [25,44]. What is especially instructive is the rapid acceleration now documented in the Western Pacific, namely, South Korea, Japan, and coastal China above all. Industrialization and dietary shift are clearly potent drivers that can reshape cancer epidemiology within a generation, consistent with the birth cohort effect described by Downham et al. [26,32]. In several countries, individuals born in the 1990s now face a fourfold higher lifetime risk of EOCRC than those born in the 1950s, a shift that has occurred over barely two generations and underscores how quickly environmental exposures can override background genetic risk.

The faster rise of rectal cancer compared with colon cancer in younger adults carries direct clinical implications. Rectal cancer treatment typically demands a multimodal approach, neoadjuvant radiotherapy, surgery, and adjuvant chemotherapy, with a toxicity profile that is especially burdensome for younger patients who may not yet have completed their families and who face decades of living with the long-term consequences of pelvic radiation and surgery, including sexual and bowel dysfunction [42]. Accelerating rates of rectal EOCRC therefore call not only for earlier detection but for treatment frameworks that explicitly account for the age-specific priorities and long-term quality-of-life concerns of younger patients.

Our risk factor analysis translates a broad literature into a ranked, quantified framework that can directly inform both individual clinical decisions and population-level strategy. The dominant contribution of family history (OR: 5.90) and IBD (OR: 4.43) is not new, and these findings have already shaped surveillance guidelines calling for earlier and more frequent colonoscopy in these groups [45]. However, it is worth emphasizing that hereditary and established medical risk factors explain only a minority of cases. Around 25-30% of EOCRC patients carry a family history that would have triggered earlier surveillance; the remaining 70-75% have no pre-existing flags and are diagnosed sporadically [38,46]. It is in this large sporadic group that modifiable exposures appear to take on the greatest epidemiological significance, though the observational nature of the underlying data precludes causal inference.

The prominence of sugar-sweetened beverages (OR: 1.55), processed meat (OR: 1.53), obesity (OR: 1.52), and Western dietary patterns (OR: 1.43) as the leading modifiable exposures are consistent with the dietary shifts that have accompanied industrialization and globalization and are biologically plausible, though these estimates derive from observational studies and residual confounding cannot be excluded. These eating patterns disrupt gut microbiome composition, sustain a state of chronic low-grade inflammation, drive insulin resistance, and may accelerate the adenoma-to-carcinoma transition, all pathways with plausible mechanistic links to earlier-onset malignancy [47-49]. The gut microbiome appears to sit at the intersection of these dietary risk factors and colorectal carcinogenesis. Dysbiosis signatures, particularly enrichment of *Fusobacterium nucleatum* and depletion of short-chain fatty acid-producing commensals, are now increasingly reported in EOCRC tumor microenvironments [50,51].

Although several modifiable exposures demonstrated statistically significant associations, these effect sizes should not be interpreted as direct measures of population-level burden. Population-attributable fractions depend on both the magnitude of association and the prevalence of exposure in the underlying population. Because exposure prevalence and harmonized attributable-risk data were inconsistently reported across the included studies, pooled population-attributable fractions were not estimated.

The independent associations of smoking (OR: 1.44) and alcohol (OR: 1.41) fit well with established carcinogenic mechanisms, DNA adduct formation, oxidative stress, and epigenetic disruption, and are not unexpected. What is more sobering is that tobacco and alcohol use have been rising among young adults in numerous low- and middle-income countries even as they decline in wealthier nations. This divergence may be an important, under-recognized driver of the emerging EOCRC burden in regions that were previously considered low risk [41].

The projection figures deserve to be taken seriously. On a business-as-usual trajectory, EOCRC incidence will reach 7.05 per 100,000 person-years globally by 2030, and the disease will claim 11% of all colon cancer diagnoses and nearly a quarter of all rectal cancer diagnoses. Because EOCRC strikes in the second through fifth decades of life, the human cost extends well beyond cancer itself; it falls on parents, partners, and wage-earners, and the economic and psychological ripple effects are correspondingly large [40,50-55].

Strengths

This analysis has several notable strengths. The search was comprehensive across three major databases, the geographic scope was broad, quality appraisal was systematic, subgroup and sensitivity analyses were prespecified rather than post hoc, secondary literature was excluded from quantitative analyses to reduce double-counting, exposure categories were harmonized a priori, and reporting followed PRISMA 2020 throughout. Extending the literature search through the end of 2025 allowed us to capture recent data in a rapidly evolving field.

Limitations of the Included Evidence

Several limitations of the included evidence should be considered. First, substantial heterogeneity remained despite subgroup and sensitivity analyses, limiting the interpretability of a single pooled summary estimate. Second, registry completeness, standard populations for age standardization, and crude versus age-standardized incidence reporting varied across studies. Third, most exposure-association data came from observational designs and may be affected by residual confounding, recall bias, measurement bias, lifestyle clustering, and socioeconomic confounding. Fourth, pooled ORs, RRs, and HRs were interpreted as approximate relative associations rather than causal effects. Finally, because the certainty of evidence was low for the primary incidence estimate and leading modifiable exposure associations, pooled estimates should be interpreted as epidemiologic summaries rather than definitive causal estimates.

Limitations of the Review Process

Limitations of the review process also warrant mention. The threshold of at least 100 EOCRC cases may have excluded informative studies from underrepresented regions. Preprint servers were not searched, and conference abstracts were excluded unless sufficient peer-reviewed full-text data were available. Although one reviewer performed initial extraction and a second independently verified all items, fully independent dual extraction may reduce extraction error in future updates. Finally, projections assume continuation of observed trends and do not account for future changes in screening uptake, diet, obesity prevalence, diagnostic intensity, or registry capture.

## Conclusions

EOCRC is increasingly recognized across diverse geographic settings and should no longer be viewed as a concern limited to historically high-income regions. This analysis makes clear that rates are rising on every continent, that the global pooled incidence is substantial and climbing, and that the trajectory points firmly upward. The Western Pacific, with the fastest documented rate of increase anywhere in the world, warrants immediate and sustained attention.

Three practical imperatives follow from these findings. First, screening policies need to keep evolving: lowering the age threshold to 45 in the United States was an important step, and there is now a serious case for exploring risk-stratified approaches in the 35-44-year age group. Second, primary prevention deserves more investment than it currently receives; interventions targeting diet quality, ultra-processed food exposure, physical activity, and obesity are biologically plausible and supported by observational epidemiologic associations, although causal estimates require further prospective evidence. Even where definitive causal estimates await prospective interventional data, the biological plausibility and cross-setting consistency of these associations provide sufficient justification to integrate these targets into public health prevention strategies. Third, every clinician seeing young adults must maintain a high index of suspicion for CRC when patients report rectal bleeding, altered bowel habits, unexplained abdominal pain, or iron deficiency anemia; delayed diagnosis is one of the most consistently reported and avoidable features of EOCRC.

Looking ahead, the field needs cancer registry investment in Africa and South Asia to close the data gap; prospective cohort studies in younger adults that track modifiable exposures longitudinally; birth cohort studies with early-life exposure data; mechanistic work to clarify how the diet-microbiome axis promotes early carcinogenesis; and, ultimately, clinical trials testing chemoprevention strategies in high-risk young people. None of this is straightforward, but none of it is beyond reach.

## Appendices

Appendix A

Database 1: PubMed/MEDLINE

Records retrieved: 3,241  |  Date run: 31 December 2025

("colorectal neoplasms"[MeSH Terms] OR "colorectal cancer"[tiab] OR

"colorectal carcinoma"[tiab] OR "colon cancer"[tiab] OR "rectal cancer"[tiab]

OR "colonic neoplasms"[MeSH Terms] OR "rectal neoplasms"[MeSH Terms])

AND

("early-onset"[tiab] OR "young-onset"[tiab] OR "early onset"[tiab]

OR

"young onset"[tiab] OR "young adults"[MeSH Terms] OR "young adult"[tiab]

OR "early age"[tiab] OR "age of onset"[MeSH Terms] OR "premature"[tiab]

OR "early age at diagnosis"[tiab] OR "under 50"[tiab] OR "below 50"[tiab]

OR "younger patients"[tiab] OR "adolescent"[MeSH Terms])

AND

("incidence"[MeSH Terms] OR "prevalence"[MeSH Terms] OR "incidence"[tiab]

OR "epidemiology"[tiab] OR "temporal trends"[tiab] OR "time trends"[tiab]

OR "secular trends"[tiab] OR "global burden"[tiab] OR "risk factors"[MeSH Terms]

OR "etiology"[MeSH Terms] OR "aetiology"[tiab] OR "population-based"[tiab]

OR "cancer registry"[tiab] OR "registries"[MeSH Terms])

Filters: None

Date range: 1990/01/01 - 2025/12/31

Database 2: Embase (Elsevier)

Records retrieved: 2,874  |  Date run: 31 December 2025

('colorectal cancer'/exp OR 'colorectal carcinoma'/exp OR 'colon cancer':ti,ab

OR 'rectal cancer':ti,ab OR 'colorectal neoplasm':ti,ab)

AND

('early onset':ti,ab OR 'young onset':ti,ab OR 'early-onset':ti,ab

OR 'young-onset':ti,ab OR 'young adult'/exp OR 'young adult':ti,ab

OR 'early age':ti,ab OR 'age of onset':ti,ab OR 'under 50':ti,ab

OR 'below age 50':ti,ab OR 'premature cancer':ti,ab OR 'adolescent'/exp

OR 'early age at diagnosis':ti,ab OR 'younger patients':ti,ab)

AND

('incidence'/exp OR 'prevalence'/exp OR 'incidence':ti,ab OR 'epidemiology':ti,ab

OR 'time trend':ti,ab OR 'secular trend':ti,ab OR 'temporal trend':ti,ab

OR 'global burden':ti,ab OR 'risk factor'/exp OR 'population based study':ti,ab

OR 'cancer registry':ti,ab OR 'disease registry'/exp OR 'annual percent change':ti,ab

OR 'joinpoint':ti,ab)

Filters: None

Date range: 1990 to 2025

Database 3: Cochrane Central Register of Controlled Trials (CENTRAL)

Records retrieved: 1,108  |  Date run: 31 December 2025

#1 MeSH descriptor: [Colorectal Neoplasms] explode all trees

#2 "colorectal cancer" OR "colon cancer" OR "rectal cancer"

   OR "colorectal carcinoma" OR "colorectal neoplasm":ti,ab,kw

#3 "early-onset" OR "young-onset" OR "early onset" OR "young onset"

   OR "young adult" OR "early age" OR "age of onset" OR "under 50":ti,ab,kw

#4 MeSH descriptor: [Incidence] explode all trees

#5 MeSH descriptor: [Prevalence] explode all trees

#6 MeSH descriptor: [Risk Factors] explode all trees

#7 "incidence" OR "prevalence" OR "epidemiology" OR "temporal trends"

   OR "global burden" OR "cancer registry" OR "time trends":ti,ab,kw

#8 #1 OR #2

#9 #3

#10 #4 OR #5 OR #6 OR #7

#11 #8 AND #9 AND #10

Date range: 1990 to Issue 12, 2025

Appendix B

**Table 9:** Standardized data extraction form. This form was piloted on five studies prior to full use and revised accordingly. One reviewer completed the extraction; a second verified all items independently. Disagreements were resolved by discussion.

Field		Extracted data/notes		
Study ID (internal)				
First author, year				
Full citation				
Country/region				
Study design		☐ Population-based registry ☐ Prospective cohort ☐ Retrospective cohort ☐ Case-control ☐ Cross-sectional ☐ Systematic review with original data		
Data source		☐ National cancer registry ☐ Hospital registry ☐ Insurance/claims data ☐ Survey/cohort ☐ GBD database ☐ Other: _____________		
Study period (years)		From: ________ To: ________		
Geographic coverage		☐ National ☐ Regional ☐ Multi-national ☐ Global		
Funding source				
Conflict of interest		☐ None declared ☐ Industry ☐ Public ☐ Not reported		
Age cut-off defining EOCRC		☐ <40 yrs ☐ <45 yrs ☐ <50 yrs ☐ <55 yrs ☐ Other: _______		
Total sample size (N)				
Number of EOCRC cases				
Crude incidence rate (per 100,000 py)				
Age-standardised incidence rate		Standard population used: _______________________		
95% confidence interval		Lower: _________ Upper: _________		
Incidence by sex (M/F)		Males: _________ Females: _________		
Incidence by subsite		Colon: _________ Rectum: _________ NOS: _________		
Incidence by age band		20-29: ___ 30-39: ___ 40-49: ___ <50 combined: ___		
Incidence by ethnicity				
Temporal trend data available?		☐ Yes (APC: _____%) ☐ Yes (AAPC: _____%) ☐ No		
Joinpoint model used?		☐ Yes ☐ No ☐ Not reported		
Trend period		From: _______ To: _______		
Risk factor name				
Exposure category/definition				
Reference category				
Effect measure reported		☐ OR ☐ HR ☐ RR ☐ Other: _______		
Effect estimate (point)				
95% confidence interval		Lower: _________ Upper: _________		
P-value				
Adjusted for confounders?		☐ Yes (list below) ☐ No ☐ Not reported		
Confounders adjusted for				
Study design for this risk factor		☐ Case-control ☐ Cohort ☐ Cross-sectional		
Sample size for this analysis		Cases: _________ Controls/total: _________		
NOS score for this study		___ / 9 stars		
#	Domain	Item	Stars (max)	Score
1	Selection	Is the case definition adequate?	★	
2	Selection	Representativeness of the cases	★	
3	Selection	Selection of controls	★	
4	Selection	Definition of controls	★	
5	Comparability	Comparability of cases and controls on the basis of the design or analysis	★★	
6	Exposure	Ascertainment of exposure	★	
7	Exposure	Same method of ascertainment for cases and controls	★	
8	Exposure	Non-response rate	★	
		TOTAL NOS SCORE	9	
		Quality grade	High ≥7/mod 4-6 /low ≤3	
